# Health workers’ adherence to malaria case management protocols in Northern Sudan: a qualitative study

**DOI:** 10.1186/s12936-024-04998-9

**Published:** 2024-05-30

**Authors:** Sahar Khalid Mohamed, Duha Khalid Mohamed, Khansaa Ahmed, Fadwa Saad, Dejan Zurovac

**Affiliations:** 1 National Malaria Control Programme, Federal Ministry of Health, Khartoum, Sudan; 2 Department of Community Medicine, Faculty of Medicine, Al-Neelain University, Khartoum, Sudan; 3 Independent Consultant, Zagreb, Croatia

**Keywords:** Malaria, Adherence, Healthcare providers, Quality of care, Sudan

## Abstract

**Background:**

Nonadherence to national standards for malaria diagnosis and treatment has been reported in Sudan. In this study, qualitative research examined the clinical domains of nonadherence, factors influencing nonadherent practices and health workers’ views on how to improve adherence.

**Methods:**

In September 2023, five Focus Group Discussions (FGDs) were undertaken with 104 health workers from 42 health facilities in Sudan’s Northern State. The participants included medical assistants, doctors, nurses, laboratory personnel, pharmacists and public health officers. The FGDs followed a semi-structured guide reflecting the national malaria case management protocol. Qualitative thematic analysis was performed.

**Results:**

Nonadherent practices included disregarding parasitological test results, suboptimal paediatric artemether–lumefantrine (AL) dosing, lack of counselling, use of prohibited artemether injections for uncomplicated and severe malaria, artesunate dose approximations and suboptimal preparations, lack of AL follow on treatment for severe malaria; and rare use of primaquine for radical *Plasmodium vivax* treatment and dihydroartemisinin-piperaquine as the second-line treatment for uncomplicated malaria. Factors influencing nonadherence included stock-outs of anti-malarials and RDTs; staff shortages; lack of training, job aids and supervision; malpractice by specialists; distrust of malaria microscopy and RDTs; and patient pressure for diagnosis and treatment. Health workers recommended strengthening the supply chain; hiring personnel; providing in-service protocol training including specialists; establishing external quality assurance for malaria diagnosis; and providing onsite supportive supervision and public health campaigns.

**Conclusions:**

This study revealed a broad spectrum of behavioural and systemic challenges in malaria management among frontline health workers in Northern Sudan, including nonadherence to protocols due to resource shortages, training gaps, a lack of supportive supervision and patient pressure. These insights, including health workers’ views about improvements, will inform evidence-based interventions by Sudan’s National Malaria Control Programme to improve health systems readiness and the quality of malaria case management.

**Supplementary Information:**

The online version contains supplementary material available at 10.1186/s12936-024-04998-9.

## Background

Malaria, a significant public health concern, continues to cast a shadow over Sudan’s healthcare landscape. The malaria burden in Sudan was estimated to be 3.7 million cases, and 1760 deaths accounted for 17.0% of the number of outpatients admitted and 14.7% of the total number of hospital admissions in 2021 [1, 2]. *Plasmodium falciparum* accounts for 87.6%, *Plasmodium vivax* accounts for 8.1%, and other *Plasmodium* species account for approximately 4.3% of malaria infections [2]. The country’s struggle with malaria is further complicated by the COVID-19 pandemic [3], the persistent spectre of war [4] and the ensuing internal displacement of populations [5]. In this fragile environment, effective evidence-based management of malaria cases is paramount, safeguarding the well-being of communities and alleviating the economic burden associated with the disease. Sudan malaria case management standards reflect the WHO 2010 “test and treat” recommendations [6], and promote a shift from presumptive treatment of fevers to confirmed malaria diagnosis with either malaria microscopy or rapid diagnostic tests (RDT) and targeted treatment with artemisinin-based combination therapy (ACT) with appropriate weight-based dosing, drug dispensing and patient counselling [7]. Artemether–lumefantrine (AL) and dihydroartemisinin–piperaquine (DHAP) are the recommended first- and second-line artemisinin-based combinations for both *P. falciparum* and *P. vivax* uncomplicated malaria, while patients with *P. vivax* should also receive radical primaquine treatment. Regarding severe malaria management, the 2023 protocols have unambiguously recommended the use of artesunate injections and reserved parenteral quinine only when artesunate is contraindicated or unavailable [8]. Injectable artemether has been policy discontinued and banned since 2017 due to its prior irrational use [7, 9].

Despite evidence-based policies and recommendations, health worker adherence to national guidelines is the key factor determining the real-world cost-effectiveness of the “test and treat” policy for malaria [10]. Improved adherence to malaria guidelines decreases malaria mortality [11] and highlights the importance of poor quality of care as a major contributor to mortality in low- and middle-income countries [12]. The levels, trends and factors of healthcare providers’ adherence to national malaria guidelines have been studied across Africa [13–16]. Despite some improvements, adherence was found to be insufficient in many settings [17–20] with series of studies evaluating outpatient malaria case management and observing deviations from testing indications [21, 22]. Additionally, a tendency to prescribe non-recommended anti-malarials even for confirmed cases [23, 24] and provide irrational anti-malarial treatments to patients who test negative for malaria has commonly been reported [25, 26]. Furthermore, a number of studies highlighted missed opportunities in delivering timely anti-malarial treatment at healthcare facilities [27, 28]. With respect to inpatient management, suboptimal quality-of-care has also been observed. Specifically, low testing rates of febrile patients on admission, persistence of presumptive anti-malarial treatment without testing or despite negative malaria tests, lack of parasitological monitoring, use of non-recommended anti-malarials and incomplete treatments are only few of the clinical deficiencies observed [29–33].

In Sudan, the quality of malaria case management, characterized by health workers’ adherence to national protocols, has been a challenge [2]. Nonadherence to standards for diagnosing and treating uncomplicated and severe malaria has been reported in different settings [9, 34–36]. For instance, despite the availability of test and treat commodities for malaria, a national outpatient survey revealed that 67% of febrile patients tested, 64% of confirmed cases treated with ACT, 17% of test-negative patients treated for malaria, 6% of prescribed ACT patients weighed, 3% promptly administered the first ACT dose and 87%, 61% and 3% of patients, respectively, counselled on dosing, treatment completion and vomiting [9]. Inpatient management of severe malaria was less commonly evaluated, however a 20-hospital survey in Gezira State suggested that only 54% of severe malaria patients received the correct dose and dosing regimen [36].

Quantitative research can estimate the magnitude of the problem, but there is a need for qualitative research to understand the subtle factors and provide more nuanced recommendations for policy implementers to improve adherence. In the absence of such data in Sudan, this qualitative study embarks on an exploration of nonadherence among healthcare providers to malaria case management protocols in the Northern State of Sudan. Delving into the intricate web of healthcare practices, this research endeavours to unearth factors undermining adherence, as perceived by the very individuals entrenched in the frontline of healthcare delivery. Furthermore, it seeks to harness their collective wisdom by capturing their recommendations, thereby paving the way towards enhanced adherence.

## Methods

### Study design

This study utilized a qualitative research design with focus group discussions (FGDs) to explore healthcare providers’ adherence to national malaria case management protocols, specifically to identify nonadherent practices, factors influencing such practices, and gather health workers’ views on how to improve adherence to national protocols.

### Study area

Northern State, one of Sudan’s 18 states, covers 348,765 km, with Dongola as its capital. Comprising seven localities, it is home to an estimated population of 1,511,442 primarily residing in rural areas along the River Nile [37]. Malaria transmission is hypoendemic, and the Sahara Desert is constrained, with an estimated incidence of 175 cases per 1000 population [37]. The selection of Northern State for the study area involved the delivery of in-service malaria case management training for health workers, an implementation platform conveniently used for the conduct of FGDs prior to training sessions. The state hosts 314 health facilities and 2345 healthcare providers distributed across various levels of care, including family health units, primary health centres, primary hospitals, and secondary and tertiary facilities [38]. At primary healthcare (PHC) facilities, RDTs serve as the exclusive diagnostic method, especially at the family health units, where the lower PHC level is operated by medical assistants (clinical practitioners with basic medical training). Health centres, representing the higher PHC levels, provide malaria microscopy services conducted by medical laboratory technologists and outpatient services provided by medical doctors. Malaria microscopy is also available at laboratories in local, general, referral, and teaching hospitals, encompassing secondary and tertiary levels of care, where outpatient and inpatient curative services are overseen by medical doctors [39]. Malaria services are supervised by the Federal and State Ministries of Health, which also oversee tasks such as updating protocols and providing training on them. The Ministry of Health also ensures the provision of essential supplies, including malaria RDTs and anti-malarial medications, most of which are free of charge. However, malaria microscopy services are still subject to charges. In 2023, a significant increase in population and healthcare providers occurred with the influx of internally displaced people, many of whom were originally from regions with high malaria transmission [40, 41].

### Study participants

**Fig. 1:**
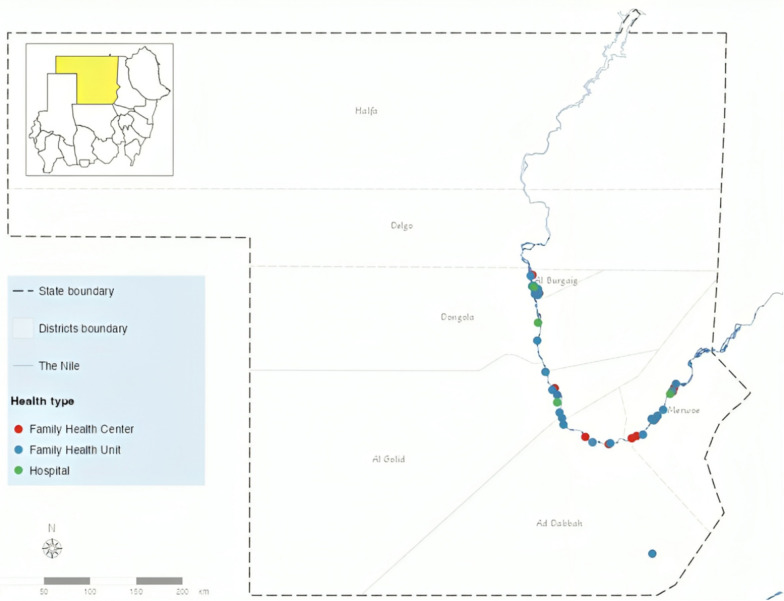
Map of Sudan showing Northern State and participant’s health facilities along the River Nile

**Fig. 2:**
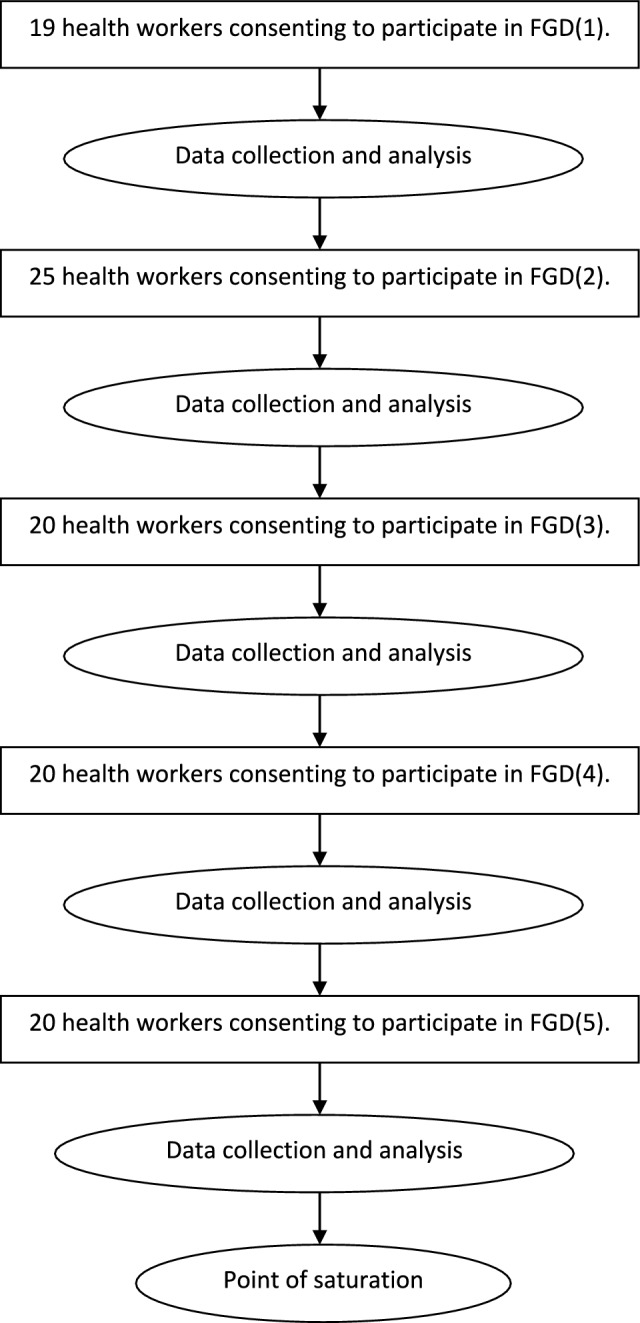
Schematic flow of the sampling process

A total of five FGDs were held at Primary Health Care Directorate venues in five out of seven localities within Sudan’s Northern State in September 2023. Healthcare providers from 42 health facilities participated in the FGDs (Fig. 1). The participants, who had diverse professional backgrounds, were chosen by the state and locality malaria control programmes to attend orientation training sessions (total of 8 training sessions for 200 health workers) on the updated malaria case management protocol [8]. The FGDs included all health workers attending an orientation session, and all study participants provided informed consent. The point of saturation through iterative data analysis after each FGD was reached after the completion of five FGD sessions, which included 104 participants in total (Fig. 2).

### Data collection

At the initiation of the orientation sessions, the collection of demographic data were collected, encompassing participants’ occupations, gender, and workplace locations. FGDs were purposefully directed toward understanding healthcare providers’ adherence to the national case management protocol, unravelling factors influencing nonadherence, and soliciting recommendations to enhance adherence. Each session of these FGDs was conducted in the Arabic language, spanning approximately 2 to 3 h, punctuated with breaks and composed of a total of 104 healthcare providers and 19 to 25 participants at each FGD. The manuscript author (SKM), following semistructured guide tailored to reflect on the study objectives on adherence to malaria case management protocols, factors affecting adherence, and recommendations to improve adherence [8], moderated the discussions. The guide, with respect to the management of uncomplicated malaria, included inquiries into participants’ knowledge regarding malaria symptoms, diagnostic procedures, first- and second-line treatments, dosage specifics, and patient counselling. Regarding severe malaria management, the guide comprised inquiries on symptoms, signs, laboratory findings, diagnostic criteria, initial management strategies, treatment locations, dosage calculations, drug preparation and administration techniques, treatment duration, discharge protocols, and second-line management. Additional questions delved into the diagnosis and management of malaria in pregnant women. Prior to its use, the guide was pretested with health workers not involved in the study (Additional file 1). Finally, participant responses prompted exploration of the underlying causes of nonadherence to the protocols, eliciting valuable suggestions to improve the training at which focus group discussions were conducted and to provide potential improvement recommendations to the Federal Ministry of Health. During the discussions, the moderator preserved neutrality and ensured that all participants felt comfortable and similarly engaged. Simultaneously with discussions, notes were taken in Arabic language, which subsequently underwent dual forward translation by two investigators (SKM and DKM) into English and transcription for comprehensive analysis.

### Data analysis

Qualitative thematic analysis was conducted [42]. It comprised seven sequential stages leveraging transcripts from FGDs and handwritten notes. Initially, all transcripts and notes were collected. Subsequently, the researcher thoroughly reviewed sample files, identifying words, phrases, or sentences pertaining to adherence to malaria diagnosis and treatment protocols. Following this, coding categories were developed using a deductive approach, with the protocol serving as the analytical framework. The fourth stage involved the coding of all files and text. The fifth stage encompassed scrutiny of the consistency in code, subthemes, and theme utilization. The sixth step entailed the interpretation of themes, drawing inferences from observed patterns, relationships, and codes, subthemes, and theme attributes. Finally, the seventh stage encompassed the presentation of findings, complemented by supporting quotes and verbatim quotes. A total of four to five themes was identified answering the research questions on healthcare providers’ adherence, factors and recommendations (Additional file 2).

## Results

### Characteristics of participants

Of 104 participants across five FGDs, most of them were males (68.2%) and most worked at the primary health care facilities (75.5%). The number of participants ranged between FGDs from 19 in Al-Golead to 25 in Dongola locality (Table 1). Healthcare providers from various backgrounds participated in the study, most commonly medical assistants (59; 56.7%), followed by doctors (13; 12.5%), public health officers (13; 12.5%), nurses (9; 8.7%), laboratory personnel (7; 6.7%) and pharmacists (3; 2.9%).

**Table 1:** Distribution of participants by occupation and FGDs

Occupation	Locality				
	AL-Golead (FGD 1)	Dongola (FGD 2)	Al-Dabba (FGD 3)	AL-Burgaig (FGD 4)	Marawi (FGD 5)
Pharmacist	0	2	0	0	1
Medical assistant	9	18	10	10	12
Nurse	2	0	2	2	3
Doctor	5	2	1	2	3
Laboratory staff	0	0	5	1	1
Public health officer	4	3	2	4	0
Total	19	25	20	20	20

### Nonadherence to national malaria protocols

Four main themes reflecting health worker clinical practices nonadherent with national malaria case management protocols were identified in the analysis (Table 2). These included malaria diagnosis practices based on parasitological tests and clinical practices related to the treatment of uncomplicated malaria, severe malaria and patients harbouring *P. vivax* and mixed malaria infections.

**Table 2:** Schematic presentation of nonadherence themes and subthemes

Themes	Subthemes
1. Nonadherence to malaria diagnosis based on parasitological tests	1.1 Presumed diagnosis of uncomplicated malaria 1.2 Presumed diagnosis of severe malaria
2. Nonadherence to uncomplicated malaria treatment	2.1 Nonadherence to the paediatric AL dosages 2.2 Lack of AL counselling practices 2.3 Nonadherence to the second-line recommendations 2.4 Irrational use of injectable anti-malarials
3. Nonadherence to severe malaria treatment	3.1 Use of prohibited artemether injections 3.2 Lack of weight-based artesunate dosing 3.3 Poor injectable artesunate preparation and disposal practices 3.4 Lack of AL follow on treatment and discharge on artesunate injections
4. Nonadherence to *P. vivax* and mixed infection treatments	4.1 Unknown risks and incorrect dosage schedules of primaquine 4.2 Dismissal of mixed infection test results

#### Nonadherence to malaria diagnosis based on parasitological tests

Universal testing of fevers and targeted anti-malarial treatment is the backbone of malaria case management in Sudan. Healthcare providers reported common ordering of a parasitological test upon suspecting malaria but highlighted distrust in the test results and many reported practicing presumed malaria diagnosis. Specifically, regarding uncomplicated malaria, several health workers reported that patients with negative test results are still diagnosed presumptively and treated for malaria:

*“Sometimes the test result comes negative for malaria, I test other causes of fever, and if all of them come negative I presume that patient has malaria” (Medical assistant)*

Higher cadres of health workers also reported presumptive treatment practices, as medical officer managing hospital outpatients similarly observed.

*“After excluding other causes of fever, even if the parasitological test is negative I treat patients as malaria and prescribe Coartem” (Medical doctor)*

Regarding the management of severely ill patients, most health workers did not mention parasitological testing and reported that anti-malarial treatment is the standard management for severe disease, as observed by medical officer working at a secondary hospital.

*“Febrile comatose patients are treated with broad spectrum antibiotic, antiviral therapy and anti-malarial treatment usually quinine, this is an umbrella approach commonly used” (Medical doctor)*

#### Nonadherence to uncomplicated malaria treatment

The AL treatment of uncomplicated malaria was highly accepted by health workers, and most participants were knowledgeable about adult doses and schedules, particularly about the importance of the second dose administration 8 h after the first dose and the third dose administered 24 h after the first dose. However, some healthcare providers have shown little knowledge about paediatric dosage schedules potentially resulting in the underdosing and overdosing of malaria patients, as demonstrated by medical assistant practicing at outpatient health centre.

*“From five to ten kilograms that is one tab of Coartem, from ten to twenty that is two tabs of Coartem, from twenty to thirty that is three tabs of Coartem” (Medical assistant)*

Most health workers during the FGDs acknowledged a lack of AL dispensing and counselling knowledge on the administration of the first dose under observation, AL dosing with a fatty meal, repeating the dose if the patient vomited within 30 min and stressing the point of taking the dose as prescribed. Some, however, highlighted that forgetfulness and unclear counselling responsibilities between different health worker cadres encountering malaria patients within health facilities contribute to poor practices. Hospital doctor and pharmacist echoed these observations.

*“I try my best to counsel all the patients but sometimes I forget” (Medical doctor).*

*“Usually we do not counsel patients and we presume that their doctor told them how to take the drug” (Pharmacist)*

Oral administration of dihydroartemisinin–piperaquine is the recommended second-line treatment for uncomplicated malaria in Sudan. However, its availability is scarce, dosing knowledge is low and health workers do not prescribe it. A healthcare provider humorously remarked on DHAP:

*“DHAP (dihydroartemisinin–piperaquine) is as rare as gold (referring to the literal meaning of Dhap in Arabic), and it is nowhere to be found” (Medical assistant)*

In this context, patients considered to have AL treatment failure are often prescribed artesunate injections reserved for severe malaria—the practice justified by the scarcity and unavailability of DHAP in the public and private sectors, as highlighted by the hospital doctor:

*“Patients may present with recurrent malaria even after taking Coartem, DHAP is not available, we have no other choice but to prescribe artesunate injections” (Medical doctor)*

Finally, despite high acceptance of AL by health workers, healthcare providers at lower levels of care (PHCs and family health units) reported the use of artemether injections as a treatment for uncomplicated malaria upon patient request:

*“Sometimes the patient refuse it when I prescribe them tabs and insist that they want the oily injections (known name for artemether injections in Sudan) and I have no other choice but to prescribe it to them” (Medical assistant)*

#### Nonadherence to severe malaria treatment

Health workers reported several treatment practices nonadherent to protocols for the management of severe malaria. Such practices spanned from the selection of non-recommended parenteral anti-malarial treatments to the lack of weight-based dosing; poor parenteral solution preparation, administration and disposal; and compromised completion of follow on ACT treatment after parenteral therapies. Participants acknowledged the frequent use of artemether injections, partly due to the patients’ pressure but also due to a lack of understanding of why artemether was prohibited while remaining available on the market. Most significantly, continued artemether use by consultants and specialists acting as supervisors and role models for front-line clinicians further undermined the treatment policy, as clearly stated by several participants of different cadres:

*“I know we should not use artemether injections, but I should do what my boss says” (Medical doctor)*

A medical assistant added,

*“When a specialist prescribes artemether injections, I find myself wondering, who am I not to do the same?”*

Most participants acknowledged not weighing adult patients but uniformly administering a 120 mg dose corresponding to a single vial of artesunate, which in turn, based on artesunate dosing recommendations of 2.4 mg/kg, results in dosing needs for patients weighing 50 kg. A lack of weighing practice in adults was well observed by the hospital doctor:

*“Usually we prescribe 120 mg to all adults, we don’t weigh them and calculate the dose accordingly, weight dependant dosage are common in paediatrics but not the common in adults” (Medical doctor)*

While weighing was probably more common in children, some healthcare providers acknowledged that the lack of weight-based artesunate dosing may also occur among paediatric patients:

*“I usually weigh the child before prescribing any medication not just malaria medications, but sometimes, when the load is heavy in the ER or the clinic I just estimate the child’s weight” (Medical doctor)*

Most participants reported uncertainties about how to prepare and administer injectable artesunate, particularly clinicians whose responsibility was related to prescribing artesunate but not to the preparing and administering of parenteral therapy:

*“We don’t prepare artesunate since it’s a nurse responsibility, but when we find ourselves in a position to do this, when nurses aren’t available, we check the directions in the box, but we weren’t trained on this before” (Registrar)*

Nurses were, however, less comfortable with IM artesunate preparations as well as with determining the number of vials needed for preparation, preparing solutions other than 120 mg for adults and disposing of unused solutions. The following remarks from hospital nurses illustrate these concerns:

*“We usually prepare IV artesunate solutions but we are not familiar with IM preparations” (Nurse)*

*“We are used to a standard dose of 120mg of artesunate injection, mixing vials to accommodate it to the patient needed dose isn’t a regular practice for us” (Nurse)*

*“We usually use the remaining solution for the next dose, we don’t dispose it and we think it will be better to save it in the refrigerator” (Nurse)*

The full course of AL should follow on IV artesunate treatment, which is administered to severe malaria patients upon admission, repeated minimally at 12 h and 24 h, and thereafter once a day until the patient can tolerate oral medicines and be discharged on oral AL therapy. Most participants, however, expressed a lack of awareness about AL follow on treatment, as clearly noted by the medical doctor:

*“I didn’t hear about this before, but if it’s in the protocol then I will do it” (Medical doctor)*

Moreover, incompletion of three minimally required IV artesunate doses at the hospital was common and discharge on injectable artesunate treatments for home administration was commonly reported:

*“I’m used to neighbours knocking my door asking me to give a patient artesunate injections at home” (Medical assistant)*

*“It’s true, I get the same neighbours asking for similar favours, and to be honest, if the patient is vitally stable we discharge him on artesunate at home” (Medical doctor)*

#### Nonadherence to *P. vivax* and mixed infection treatments

While most participants were aware of radical *P. vivax* treatment with primaquine, the scarcity of primaquine on the market contributed to the low number of prescriptions. When primaquine is prescribed, health workers often have limited knowledge about primaquine doses, glucose-6-phosphate dehydrogenase (G6PD) risks, and adjusted dosing schedules for G6PD-deficient patients, as shown below:

*“If a patient has P. vivax I prescribe them with 15 mg primaquine tabs twice daily for 2 weeks, I don’t ask specific questions to get a clue if the patient has G6PD honestly” (Medical assistant)*

*“I didn’t know about the contraindication of primaquine with G6PD, even the paediatric weight dependant dose, I’m used to prescribe 7.5 mg tabs once per day for 14 days to all paediatric patients” (House officer)*

Moreover, heath workers were less familiar with the possibility of mixed infections, particulary with test result interpretations, as observed by medical assistant below:

*“Sometimes the RDT shows both P. falciparum and P. vivax, I don’t know what the meaning of this is, I presume that the RDT is not working well and I diagnose the patient with having P. falciparum infection” (Medical assistant)*

### Factors influencing nonadherence to protocols

Four broad themes describing factors influencing nonadherence to case management protocols were identified (Table 3). These included lack of commodities and shortage of human resources, poor knowledge of health workers about case management protocols, distrust in parasitological test results and patient pressure on modalities of clinical malaria management.

**Table 3:** Schematic presentation of themes and subthemes related to nonadherence factors

Themes	Subthemes
1. Lack of commodities and shortage of human resources	1.1 Lack of anti-malarials and RDTs 1.2 Lack of weighing scales 1.3 High workload and insufficient human resources
2. Lack of training and lack of continuous support for protocol adherence	2.1 Lack of job aids and protocol booklets 2.2 Lack of continuous training especially higher level cadres 2.3 Lack of onsite supportive supervision
3. Distrust in parasitological test results	3.1 Distrust in malaria microscopy 3.2 Distrust in malaria RDTs
4. Patient pressure	4.1 Patient pressure about malaria diagnosis 4.2 Patient pressure about malaria treatment

#### Lack of commodities and shortage of human resources

The absence of diagnostics and medicines precludes adherence to case management protocols. Common stock-outs of anti-malarials and RDTs at public health facilities, the reliance upon the private sector for costly purchases and the nearly universal absence of primaquine and DHAP from the market have been commonly reported by most participants, as illustrated by the hospital doctor:

*“Malaria medications and RDTs aren’t always available in the hospital, most can be found in the private pharmacies and laboratories, except for primaquine and DHAP, they are hard to find” (Medical doctor)*

The lack of commodities is limited not only to medicines and diagnostics but also to basic facility equipment, such as weighing scales, which are required to implement appropriate weight-based dosing of patients. The majority of participants seconded the frustrations highlighted by the medical assistant:

*“I don’t have a weight scale in my centre, how am I supposed to adopt a weight dependent approach?!” (Medical assistant)*

Moreover, even when commodities are available a major effort may be required to adhere to the protocols due to high patient workloads in the face of staff shortages, as also well observed by the hospital nurse:

*“Now I understand I should prepare quinine dose just before administration, but I’m working alone and usually I have many patients to observe, this will definitely be a challenge” (Nurse)*

#### Lack of training and lack of continuous support for protocol adherence

Participants commonly attributed nonadherence to a lack of knowledge and information transfer through interventions such as regular in-service training and supportive supervision, including the delivery of reminders about good practices. For instance, the role of job aids such as poster wall charts and protocol booklets was repeatedly emphasized by the participants:

*“If I have a poster in my centre it will remind me if I forget” (Medical assistant)*

This was similarly echoed by medical doctors in hospital settings:

*“Posters help us train our house officers and as he said it helps reminding us if we forget” (Registrar)*

With respect to more formal capacity building, in-service training has been acknowledged as a valuable intervention in transmitting knowledge and enhancing case management readiness for all health workers, however, it appears that consultants and specialists have not been sufficiently reached:

*“When prestigious consultants treat malaria differently than us, we lose patients trust” (Medical assistant)*

Finally, most participants acknowledged that the lack of supportive supervision to address the availability of commodities and provide on-job support and problem solving for front-line health workers may further facilitate nonadherent practices:

*“You ask us to do things but at the same time you don’t avail the needed requirements for us, why don’t you come and see the setting at which we are practicing first?” (Medical assistant)*

#### Distrust in parasitological test results

Treatment nonadherent to malaria test results due to the distrust of malaria microscopy and doubtful quality of laboratory services was a recurrent theme among the participants:

*“Sometimes a patient presents with fever, we exclude all other causes, but if the microscopic test results come back negative from the lab, we do not trust, we manage as malaria, and we observe dramatic improvement in patients.” (Medical officer)*

Notably, clinicians’ distrust in malaria microscopy has been exclusively directed towards negative test results, as well remarked by laboratory specialist:

*“If I provide a negative result, the healthcare provider will not trust me, and they will send the patient to another lab that gives them positive results” (Laboratory specialist)*

Discontinuation of external quality assurance systems for laboratories and a lack of recent refresher training for malaria microscopists does not mitigate the distrust in test results:

*“Previously the program used to take slides from our lab for verification and provide training if the staff is giving wrong readings, but now this is not happening!” (Laboratory specialist)*

Finally, similar levels of distrust were reported with respect to malaria RDT results. Furthermore, much of the confusion was reported in the PHC facilities with regard to the appropriate performance and interpretation of RDTs produced by different companies:

*“The RDTs I have only gives me a negative result no matter what, I suspect it might be due to storage conditions” (Medical assistant)*

*“I don’t understand how to use RDTs, some companies require waiting for 15 min, some for more or less, having RDTs from different companies each time is tiring and exhausting because I’m already overworked” (Medical assistant)*

#### Patient pressure

In addition to health worker and health system factors, patient pressure may also influence health worker adherence to malaria case management protocols and alter health worker clinical practices. For instance, some health workers illustrated how patients may influence diagnosing practices for malaria:

*“When you ask the patient what are you complaining from he says malaria! And when I try to explain that I’m asking about the symptoms he says I know my malaria just order me the test” (Medical doctor)*

*“Patients insist it’s malaria even if the test is negative” (Medical assistant)*

The other participants illustrated how patients may influence treatment practices:

*“Patients insist its malaria even if the test is negative, they insist on being treated for malaria and if I didn’t write the drug they will buy it themselves” (Medical assistant)*

*“Sometimes the patient have malaria but he demands injectable treatment although they should just take tabs, some insist on taking artemether injections even after I counsel them about it” (Medical doctor)*

### Health worker recommendations to improve adherence

The participants provided valuable insights into what could be done to address the identified challenges and improve adherence to malaria case management protocols, as summarized in Table 4.

**Table 4:** Schematic presentation of recommendation themes and subthemes

Theme	Subtheme
1. Ensuring availability of case management commodities	1.1 Ensuring availability of malaria diagnostics 1.2 Ensuring availability of malaria medicines
2. Improving balance between human resources and workload	2.1 Hiring more healthcare providers 2.2 Advocating for a balanced workload and working hours
3. In-service training on case management protocols	3.1 Training focusing on specialists and consultants 3.2 Understanding the prohibition of artemether injections
4. Quality assurance for diagnosis and supervision activities	4.1 External quality assessments for malaria diagnosis 4.2 Supervision of public health facilities 4.3 Supervision of private pharmacies
5. Health promotion campaigns	5.1 Promotion of parasitological malaria diagnosis 5.2 Promotion of rational anti-malarial use

#### Ensuring the availability of case management commodities

Health workers emphasized the responsibility of the Ministry of Health to strengthen the effective supply chain for malaria commodities and ensure the universal, continuous and affordable availability of malaria diagnostic and treatment commodities. Such steps would present a basic prerequisite for adherence improvements:

*“If RDTs are free and available we can take a step towards accurate diagnosis of malaria” (Medical assistant)*

*“I don’t prescribe primaquine for patients with P. vivax because it’s not available, same goes for DHAP” (Medical assistant)*

#### Improving the balance between human resources and workload

Quality of care is dependent on adequate human resources and calls for additional personnel to balance heavy workloads were the theme of transpiring discussions across the cadres of participants:

*“Now I understand I should prepare quinine dose just before administration, but I’m working alone and usually I have many patients to observe, this will definitely be a challenge, I need more people to work with me” (Nurse)*

*“Being overworked and not have much sleep can alter your cognitive skills! This is scientifically proven! The Ministry of Health should decrease our workload because this isn’t just affecting malaria management, this is affecting all patient’s health outcome” (Medical doctor)*

#### In-service training on case management protocols

Participants emphasized the importance of continuous training for all health workers. Recognizing the influential role played by specialists and consultants in hospital settings, they also underscored the importance of targeted training and interventions for these professionals:

*“Now, I know the updated protocol, but my boss doesn’t, I may adhere to it in my private outpatient clinic but in the hospital it’s the consultant’s decision, not mine, you have to orient him too with the protocol” (Medical officer)*

Moreover, whether implemented during training or through separate communication channels, health workers expected clarifications about the reasons for artemether injection prohibitions:

*“The consultant in my unit is prescribing artemether injection, I do too since I don’t understand why is it prohibited at the first place, if you are afraid of the resistance because of the mono-therapies, why would you put artesunate injection in the protocol?” (Medical doctor)*

#### Quality assurance for malaria diagnosis and supervision activities

Most of the participants called for external quality assessments at public laboratories through onsite supervisory visits for malaria microscopy and RDTs:

*“I don’t trust the lab results, supervising the lab and making sure it’s well equipped and its staff is qualified will be great” (Medical doctor)*

*“The RDTs I have only give a negative result, I would like if the Ministry of Health came and saw if it’s working or not or if its storage is well or not” (Medical assistant)*

Some hospital clinicians further emphasized MoH supervision at public pharmacies and laboratories with respect to the enforcement of free policy for government procured RDTs and artesunate vials:

*“When artesunate is available in the hospital, only the first dose is free, the patient has to buy other doses from the private pharmacy, you should talk to them to make sure they provide all doses for free” (Medical officer)*

Finally, participants highlighted the importance of extending supervision to the private pharmacies to ensure the delivery of quality products and compliance with artmether injection prohibition:

*“If artemether is prohibited then why the government doesn’t enforce its prohibition by law? Supervise the private pharmacies!” (Medical assistant)*

*“Artesunate injection isn’t available in the public hospital, patients buy it from the private pharmacies and the quality of the drug available isn’t good” (Medical doctor)*

#### Health promotion campaigns

Participants emphasized that public campaigns are necessary to promote malaria diagnosis based on parasitological test results and to increase the understanding of the rational use of anti-malarial medicines:

*“Patients and co-patients don’t trust the parasitological test and insist on the malaria diagnosis, the concept “I know my Malaria” needs to be fought” (Medical assistant)*

*“Patients insist on being treated for malaria even they don’t have malaria, and if they do they insist on being treated through injections rather than tabs, let alone those who request artemether injections” (Medical doctor)*

## Discussion

Sudan health workers from Northern State revealed a broad spectrum of protocol nonadherent malaria case management practices, factors influencing such practices and recommendations of relevance for policy implementers to improve the adherence to and quality of malaria care. The problem of malaria diagnosis transpired throughout the study. Stock-outs of diagnostic commodities such as RDTs, distrust of malaria microscopy, but also RDTs, limited in-service training and lack of supportive supervision for laboratories and clinicians have been highlighted in this study, as similarly observed in various settings across Africa [26–29, 43, 44]. More recently, compliance with test negative test results and the rational use of anti-malarials have improved in other countries [14, 25], and many of these achievements have been attributed to the implementation of quality assurance programmes for malaria diagnosis supported with systems readiness and case management monitoring [15, 45]. In Sudan, as also suggested by the study findings, the quality assurance programmes traditionally targeting malaria microscopy should be re-established but also expanded to include RDTs through the onsite supervision of non-laboratory personnel, in line with recently developed quality assurance guidelines [46]. To mitigate human resource issues, outpatient use of RDTs for initial febrile visits should be promoted at all levels of care, including hospitals, while complex to perform malaria microscopy should be reserved for follow-up of treatments and monitoring of parasitaemia for severely admitted patients [47, 48].

Another quality of care aspect severely compromising the effectiveness of malaria case management is nonadherence to treatment recommendations [47]. Health workers revealed several suboptimal treatment practices. First, lack of patients’ weighing and the practice of dose approximations, the clinical deficiencies not unique to Sudan [29, 33], are widespread and inevitably result in overdosed and underdosed treatments. Although the lack of weighing scales may provide a plausible explanation, uniformed artesunate prescriptions for adults based on a single 120 mg vial may also reflect cost considerations, ease of administration and waste minimization. Second, paediatric anti-malarial misdosing reported by health workers may not only be due to the absence of weighing scales but also due to deficient dosing knowledge. Third, suboptimal counselling practices, as similarly observed for outpatients in other settings [9, 28, 34], are important components of malaria case management, compromising the promptness of the treatment, patient adherence, cure rates, and follow-up needs [49, 50]. While acknowledged in the protocols, patient counselling has been a neglected topic and has indeed not been addressed during case management in-service trainings for health workers in Sudan [51]. Fourth, while the first-line treatment recommendations for uncomplicated malaria are highly accepted by health workers, the use of injectable artemether due to patient pressure seems to compromise the implementation of the treatment policy. This irrational treatment pattern was observed during earlier national surveys [9, 40], raised concerns about drug resistance and ultimately led to the prohibition of injectable artemether in Sudan [7]. Although the findings of this study suggest persistent artemether use for uncomplicated malaria, quantitative assessments are required to estimate the scale of this malpractice. Fifth, regarding the treatment of severe malaria, the widespread use of injectable artemether, the inferior anti-malarial choice for this category of patients, as well as incomplete ACT follow on treatments are the nonadherent aspects of care described previously [29, 30], and are perhaps the most concerning since they directly compromise patient outcomes [52, 53]. Artemether preferences, especially common among role model cadres such as consultants and specialists, might reflect a lack of updates about treatment effectiveness but also weak regulatory enforcements given the banned status of the product on the market. Finally, the recommended management of the treatment failures and radical treatment of *P. vivax* infections is uncommon in Sudan due to the very low availability of respective treatments for these special patient groups, DHAP and primaquine.

Quality improvement initiatives targeting the readiness of health systems and the adherence of health workers to evidence-based case management protocols are malaria control priorities in Sudan [36]. Reinforcement of the test and treat practices is important not only for delivering quality of care and curbing malaria mortality but also for establishing reliable disease surveillance and malaria elimination foundations to which the country, and in particular the northern states of Sudan, are aspiring to. The study health workers in the Northern State suggested several valuable interventions that concur with broad strategic plans of the National Malaria Control Program (NMCP) [37]. Quality assurance for malaria diagnosis, the programmatic intervention mentioned earlier, is one of the control priorities. Regarding the initiatives targeting clinicians, only smaller-scale, multifaceted projects have been piloted [54], while programmatic interventions focusing mainly on supply, protocols and training have had limited reach [2]. In the following years, the Sudan NMCP plans to implement a package of multifaceted, evidence-based, country-adapted, quality improvement interventions focusing on the provision of diagnostics and medicines; supportive supervision, including audit, feedback and mentorship; and in-service training coupled with monitoring and group problem solving, interventions that have been shown to have significant positive effects [55]. Clinical algorithms in the format of job aids will be integral components of the multifaceted training and supervision interventions, required to remind health workers but also policy makers about key case management standards. However, it should be emphasized that improved performance through health worker supportive interventions such as job aids, training and supervision can be realized only if basic prerequisites are in place, i.e., universal and continuous availability of “test and treat” commodities and services for malaria [56]. Finally, regular assessments of the quality of care will be established to provide reliable quantitative indicators to inform progress in health system readiness and adherence to outpatient and inpatient malaria case management protocols [9, 33, 57].

In addition to health system interventions, health campaigns have been suggested for Sudan’s Northern State to mitigate patient pressure by increasing public awareness of parasitological diagnosis and decreasing demand for injectable medicines, especially for artemether injections. Other countries reported differing effects of patient pressure on anti-malarial prescriptions [58]. In Sudan, further quantitative research is needed to assess relation between appropriate counselling, patient pressure and patient satisfaction level. Finally, while staff shortages are a pertinent health system problem in Sudan and additional hiring, as requested by health workers, is a long-term solution, more effective use of available resources such as task shifting of RDT performance is a more realistic palliative solution in the short term.

Several study limitations should be acknowledged. First, while the study findings represent Northern State health workers, they may not be generalizable to other parts of Sudan. Second, the study did not include policy makers and implementers what limited insights into adherence factors only to the recipients of the case management interventions. Third, while the social desirability bias in reporting clinical behaviour cannot be excluded, this bias appeared to be controlled given the extent of reported nonadherent practices. Finally, the identification and exploration of nonadherent topics were guided by case management protocols and not by quantitative adherence data in the study area. Future studies exploring adherence to protocols should deploy mixed methods designs.

## Conclusions

This study revealed a broad spectrum of behavioural and systemic challenges in malaria management among Northern Sudan’s frontline health workers, including nonadherence to protocols due to resource shortages, training gaps, lack of supportive supervision and patient pressure. These insights, including health workers’ views about improvements, will inform evidence-based interventions by Sudan’s National Malaria Control Programme to improve health system readiness and the quality of malaria case management.

### Supplementary Information

Supplementary Material 1.

Supplementary Material 2.

## Data Availability

The datasets used and analysed during the current study are available from the corresponding author upon reasonable request.

## References

[1] WHO (2023). World malaria report 2023.

[2] Federal Ministry of Health—Sudan. Malaria program review, 2023. National Malaria Control Program; 2023.

[3] World Bank. Socioeconomic impact of COVID-19 on Sudanese households. Central Bureau of Statistics—Sudan. December 2020. https://documents1.worldbank.org/curated/en/394981620725892919/pdf/Socioeconomic-Impact-of-COVID-19-on-Sudanese-Households.pdf. Accessed 24 Feb 2024.

[4] WHO. Three months of violence in Sudan: health hanging in the balance. WHO—Regional Office for Africa; 2023. https://www.afro.who.int/news/three-months-violence-sudan-health-hanging-balance. Accessed 10 Dec 2023.

[5] WHO. Health needs heighten as Sudan conflict displaces millions of people. WHO—Eastern Mediterranean region; 2023. http://www.emro.who.int/media/news/health-needs-heighten-as-sudan-conflict-displaces-millions-of-people.html. Accessed 10 Dec 2023.

[6] WHO. Guidelines for the treatment of malaria, 2nd edition. Geneva: World Health Organization; 2010. https://www.paho.org/en/documents/guidelines-treatment-malaria-second-edition-2010. Accessed 10 Dec 2023.

[7] Federal Ministry of Health—Sudan. Sudan malaria case management protocol. National Malaria Control Programme; 2017.

[8] Federal Ministry of Health—Sudan. Sudan malaria case management protocol. National Malaria Control Program; 2023.

[9] Abdelgader I, Elmardi KA, Githinji S, Zurovac D, Snow RW, Noor AM (2012). Quality of malaria case-management in Sudan. BMC Public Health.

[10] Lubell Y, Reyburn H, Mbakilwa H, Mwangi R, Chonya K, Whitty CJM (2007). The cost-effectiveness of parasitologic diagnosis for malaria-suspected patients in an era of combination therapy. Am J Trop Med Hyg.

[11] Biai S, Rodrigues A, Gomes M, Ribeiro I, Sodemann M, Alves F (2007). Reduced in-hospital mortality after improved management of children under 5 years admitted to hospital with malaria: randomised trial. BMJ.

[12] Kruk M, Gage A, Joseph N, Danaei G, García-Saisó S, Salomon J (2018). Mortality due to low-quality health systems in the universal health coverage era: a systematic analysis of amenable deaths in 137 countries. Lancet.

[13] Amboko B, Stepniewska K, Malla L, Machini B, Bejon P, Snow RW (2021). Determinants of improvement trends in health workers’ compliance with outpatient malaria case-management guidelines at health facilities with available “test and treat” commodities in Kenya. PLoS ONE.

[14] Amboko B, Stepniewska K, Macharia PM, Machini B, Bejon P, Snow RW (2020). Trends in health workers’ compliance with outpatient malaria case-management guidelines across malaria epidemiological zones in Kenya, 2010–2016. Malar J.

[15] Amboko B, Stepniewska K, Machini B, Bejon P, Snow RW, Zurovac D (2022). Factors influencing health workers’ compliance with outpatient malaria ‘test and treat’ guidelines during the plateauing performance phase in Kenya, 2014–2016. Malar J.

[16] Bawate C, Callender-Carter ST, Nsajju B, Bwayo D (2016). Factors affecting adherence to national malaria treatment guidelines in management of malaria among public healthcare workers in Kamuli District, Uganda. Malar J.

[17] Muhindo Mavoko H, Ilombe G, Inocêncio da Luz R, Kutekemeni A, Van Geertruyden J-P, Lutumba P (2015). Malaria policies versus practices, a reality check from Kinshasa, the capital of the Democratic Republic of Congo. BMC Public Health.

[18] Salomão CA, Sacarlal J, Chilundo B, Gudo ES (2015). Prescription practices for malaria in Mozambique: poor adherence to the national protocols for malaria treatment in 22 public health facilities. Malar J.

[19] Bulafu D, Nagawa Tamale B, Ninsiima LR, Baguma JN, Namakula LN, Niyongabo F (2023). Adherence to malaria treatment guidelines among health care workers in private health facilities in Kampala’s informal settlements, Uganda. PLoS Global Public Health.

[20] Baraka V, Nhama A, Aide P, Bassat Q, David A, Gesase S (2023). Prescription patterns and compliance with World Health Organization recommendations for the management of uncomplicated and severe malaria: a prospective, real-world study in sub-Saharan Africa. Malar J.

[21] Plucinski MM, Ferreira M, Ferreira CM, Burns J, Gaparayi P, Joao L (2017). Evaluating malaria case management at public health facilities in two provinces in Angola. Malar J.

[22] Plucinski MM, Guilavogui T, Camara A, Ndiop M, Cisse M, Painter J (2018). How far are we from reaching universal malaria testing of all fever cases?. Am J Trop Med Hyg.

[23] Bruxvoort KJ, Leurent B, Chandler CIR, Ansah EK, Baiden F, Bjorkman A (2017). The impact of introducing malaria rapid diagnostic tests on fever case management: a synthesis of ten studies from the ACT Consortium. Am J Trop Med Hyg.

[24] O’Boyle S, Bruxvoort KJ, Ansah EK, Burchett HED, Chandler CIR, Clarke SE (2020). Patients with positive malaria tests not given artemisinin-based combination therapies: a research synthesis describing under-prescription of antimalarial medicines in Africa. BMC Med.

[25] Boyce MR, O’Meara WP (2017). Use of malaria RDTs in various health contexts across sub-Saharan Africa: a systematic review. BMC Public Health.

[26] Kabaghe AN, Visser BJ, Spijker R, Phiri KS, Grobusch MP, van Vugt M (2016). Health workers’ compliance to rapid diagnostic tests (RDTs) to guide malaria treatment: a systematic review and meta-analysis. Malar J.

[27] Namuyinga RJ, Mwandama D, Moyo D, Gumbo A, Troell P, Kobayashi M (2017). Health worker adherence to malaria treatment guidelines at outpatient health facilities in southern Malawi following implementation of universal access to diagnostic testing. Malar J.

[28] Steinhardt LC, Chinkhumba J, Wolkon A, Luka M, Luhanga M, Sande J (2014). Quality of malaria case management in Malawi: results from a nationally representative health facility survey. PLoS ONE.

[29] Ojo AA, Maxwell K, Oresanya O, Adaji J, Hamade P, Tibenderana JK (2020). Health systems readiness and quality of inpatient malaria case-management in Kano State. Malar J.

[30] Signorell A, Awor P, Okitawutshu J, Tshefu A, Omoluabi E, Hetzel MW (2023). Health worker compliance with severe malaria treatment guidelines in the context of implementing pre-referral rectal artesunate in the Democratic Republic of the Congo, Nigeria, and Uganda: an operational study. PLoS Med.

[31] Achan J, Tibenderana J, Kyabayinze D, Mawejje H, Mugizi R, Mpeka B (2011). Case management of severe malaria-a forgotten practice: experiences from health facilities in Uganda. PLoS ONE.

[32] Shah MP, Briggs-Hagen M, Chinkhumba J, Bauleni A, Chalira A, Moyo D (2016). Adherence to national guidelines for the diagnosis and management of severe malaria: a nationwide, cross-sectional survey in Malawi. Malar J.

[33] Zurovac D, Machini B, Kiptui R, Memusi D, Amboko B, Kigen S (2018). Monitoring health systems readiness and inpatient malaria case-management at Kenyan County Hospitals. Malar J.

[34] Elmannan AAA, Elmardi KA, Idris YA, Spector JM, Ali NA, Malik EM (2015). Anti-malarial prescribing practices in Sudan eight years after introduction of artemisinin-based combination therapies and implications for development of drug resistance. BMC Pharmacol Toxicol.

[35] Bilal JA, Gasim GI, Abdien MT, Elmardi KA, Malik EM, Adam I (2015). Poor adherence to the malaria management protocol among health workers attending under-five year old febrile children at Omdurman Hospital Sudan. Malar J.

[36] Elnour FA, Alagib MEA, Bansal D, Farag EABA, Malik EM (2019). Severe malaria management: current situation, challenges and lessons learned from Gezira State, Sudan. Malar J.

[37] Federal Ministry of Health—Sudan. Sudan malaria strategic plan 2021–2026, update March 2023. National Malaria Control Programme; 2023.

[38] WHO. Understanding the labour market of human resources for health in Sudan. Working Paper, November 2013. https://www.who.int/publications/m/item/2013-sudan-hrh-labour-market. Accessed 15 Dec 2023.

[39] Ebrahim EMA, Ghebrehiwot L, Abdalgfar T, Juni MH (2017). Health care system in Sudan: review and analysis of strength, weakness, opportunity, and threats (SWOT analysis). Sudan J Med Sci.

[40] Federal Ministry of Health—Sudan. Sudan malaria indicator survey 2016. Directorate of Communicable and Non-communicable Diseases Control; 2017.

[41] Office for the Coordination of Humanitarian Affairs. Sudan situation report, 2 November 2023. https://www.unocha.org/publications/report/sudan/sudan-situation-report-2-november-2023-enar. Accessed 16 Dec 2023.

[42] Nowell LS, Norris JM, White DE, Moules NJ (2017). Thematic analysis: striving to meet the trustworthiness criteria. Int J Qual Methods.

[43] Hanson K, Goodman C (2017). Testing times: trends in availability, price, and market share of malaria diagnostics in the public and private healthcare sector across eight sub-Saharan African countries from 2009 to 2015. Malar J.

[44] Kuupiel D, Bawontuo V, Drain PK, Gwala N, Mashamba-Thompson TP (2019). Supply chain management and accessibility to point-of-care testing in resource-limited settings: a systematic scoping review. BMC Health Serv Res.

[45] Ashton RA, Worges M, Zeh Meka A, Yikpotey P, Domkam Kammogne I, Chanda-Kapata P (2023). Can outreach training and supportive supervision improve competency in malaria service delivery? An evaluation in Cameroon, Ghana, Niger, and Zambia. Am J Trop Med Hyg.

[46] Federal Ministry of Health—Sudan. Sudan quality assurance guide for parasitological diagnosis of malaria (draft). National Malaria Control Programme; 2023.

[47] WHO (2016). Malaria microscopy—quality assurance manual.

[48] Galactionova K, Tediosi F, de Savigny D, Smith T, Tanner M (2015). Effective coverage and systems effectiveness for malaria case management in sub-Saharan African countries. PLoS ONE.

[49] Mace KE, Mwandama D, Jafali J, Luka M, Filler SJ, Sande J (2011). Adherence to treatment with artemether–lumefantrine for uncomplicated malaria in rural Malawi. Clin Infect Dis.

[50] Talisuna AO, Oburu A, Githinji S, Malinga J, Amboko B, Bejon P (2017). Efficacy of text-message reminders on paediatric malaria treatment adherence and their post-treatment return to health facilities in Kenya: a randomized controlled trial. Malar J.

[51] Federal Ministry of Health—Sudan. Malaria case management training manual. Directorate of Communicable and Non Communicable Disease; 2023.

[52] Phu NH, Tuan PQ, Day N, Mai NT, Chau TT, Chuong LV (2010). Randomized controlled trial of artesunate or artemether in Vietnamese adults with severe falciparum malaria. Malar J.

[53] Hetzel MW, Okitawutshu J, Tshefu A, Omoluabi E, Awor P, Signorell A (2022). Effectiveness of rectal artesunate as pre-referral treatment for severe malaria in children under 5 years of age: a multi-country observational study. BMC Med.

[54] Mohammed NMA. Impact of multifaceted clinical pharmacist interventions on implementation of national malaria protocol, in Wad Madani Maternity Hospital, Gezira State, Sudan (2020). PhD Thesis—University of Gezira; July 2022.

[55] Rowe AK, Rowe SY, Peters DH, Holloway KA, Chalker J, Ross-Degnan D (2018). Effectiveness of strategies to improve health-care provider practices in low-income and middle-income countries: a systematic review. Lancet Glob Health.

[56] Bernard YM, Ahmed J, Mostel J, Ba T, Ciceron AC, Busiga M (2023). Clinical outreach training and supportive supervision quality-of-care analysis: impact of readiness factors on health worker competencies in malaria case management in Cameroon, Mali, and Niger. Am J Trop Med Hyg.

[57] Zurovac D, Githinji S, Memusi D, Kigen S, Machini B, Muturi A (2014). Major improvements in the quality of malaria case-management under the “test and treat” policy in Kenya. PLoS ONE.

[58] Chandler CI, Mwangi R, Mbakilwa H, Olomi R, Whitty CJ, Reyburn H (2008). Malaria overdiagnosis: is patient pressure the problem?. Health Policy Plan.

