# Supplementary material for: Health workers’ adherence to malaria case management protocols in Northern Sudan: a qualitative study
Source: Malar J. 2024 May 30;23:170. doi: 10.1186/s12936-024-04998-9 (PMC11137897; doi:10.1186/s12936-024-04998-9)
Supplement: Supplementary file 1 — Supplementary Material 1. [file 12936_2024_4998_MOESM1_ESM.docx]

**Focus Group Discusion guide**

*Introduction:*

- Welcome and express gratitude for participation.
- Briefly explain the purpose of the FGD: to explore current non-adherent practices in malaria case management protocols, factors influencing them, and recommendations to improve adherence. Obtain verbal informed consent and start the FGD.
- Note that this is a sensitive topic, take a non-judgmental stance, and encourage participants to freely report their practices.
- Use this guide to navigate the discussion, asking open-ended questions, and following up on reported practices. Encourage participants to share their points of view and assure them that their recommendations are important.
- Request background information about occupation, and work location.

*Non-adherent practices:*

Since this topic is sensitive, ensure that it is approached with respect, ensure all participants actively participate in the discussion, use follow-up questions to add more depth to the insights, and keep notes and attention on non-adherent practices to follow up on them.

*Diagnostic practices in adults:*

- In which patients do you suspect simple malaria?
- How do you diagnose simple malaria?
- In which patients do you suspect severe malaria?
- How do you diagnose severe malaria?
- How do you diagnose *P.*vivax malaria?
- How do you diagnose mixed infection malaria?
- Do you depend on parasitological testing to confirm it?
- What parasitological testing do you prefer, and why?
- How confident are you in the accuracy of blood films?
- How confident are you in the accuracy of RDT results?

*Treatment practices in adults:*

- Do you ever treat patients who test negative for malaria? If yes, is it more likely to disregard RDTs or microscopy test results? Is it more likely for severe or mild cases?
- How do you treat simple malaria(Include dosage calculations, treatment duration, second line management, and counseling practices) ?
- How do you treat severe malaria? (Include dosage calculations, drug preparation, administration techniques, treatment duration, discharge protocols, second line management, and counseling practices )
- How do you treat *P.*vivax malaria?
- How do you treat mixed infection malaria?
- How do you confirm treatment failure?

*Malaria in children:*

- Is diagnosing malaria in children similar to adults? If not, how is it different?
- How do you treat children with malaria?
- How do you treat children with severe malaria?
- How do you treat children with P.vivax malaria?
- How do you treat children with mixed malaria infection?

*Malaria in pregnancy:*

- Is diagnosing malaria in pregnant women similar to diagnosing it in adults? If not, how is it different?
- How do you treat pregnant women with simple malaria?
- How do you treat pregnant women with severe malaria?
- How do you treat pregnant women with *P.*vivax malaria?
- How do you treat pregnant women with mixed malaria infection?

*Factors influencing non adherent practices:*

- Are malaria commodities (RDTs, microscopy, Coartem, artesunate injections, Quinine infusions) available in your workplace?
- What influences non-adherent practices in malaria diagnosis and tratment in your opinion? (Ask this question about adults, children, and pregnant individuals.)

*Recommendations to improve adherent practices:*

- How to improve malaria diagnostic and management practices? ask this question about adults, children and pregnant.
