# Supplementary material for: Health workers’ adherence to malaria case management protocols in Northern Sudan: a qualitative study
Source: Malar J. 2024 May 30;23:170. doi: 10.1186/s12936-024-04998-9 (PMC11137897; doi:10.1186/s12936-024-04998-9)
Supplement: Supplementary file 2 — Supplementary Material 2. [file 12936_2024_4998_MOESM2_ESM.docx]

Additional file 2 :Themes and subthemes across the study questions

Non-adherence to uncomplicated malaria treatment

Non-adherent practices Factors influencing non-adherence Recommendations to improve adherence

Themes Subthemes Themes Subthemes Themes Subthemes

Health promotion campaigns

Quality assurance for diagnosis and supervision activities

Ensuring availability of malaria diagnostics

Presumed diagnosis of uncomplicated malaria

Presumed diagnosis of uncomplicated malaria

Ensuring availability of case management commodities

Non-adherence to severe malaria treatment

Non-adherence to uncomplicated malaria treatment

Non-adherence to malaria diagnosis based on parasitological tests

Non-adherence to *P.vivax* and mixed infection treatments

Non-adherence to malaria diagnosis based on parasitological tests

Lack of weighing scales

Non-adherence to the paediatric AL dosages

Irrational use of injectable antimalarials

Presumed diagnosis of severe malaria

Non-adherence to the second line recommendations

Lack of AL counselling practices

Presumed diagnosis of severe malaria

Hiring more healthcare providers

Understanding the prohibition of artemether injections

Training focusing on specialists and consultants

In-service training on case management protocols

Improving balance between human resources and workload

Lack of AL counselling practices

Non-adherence to the paediatric AL dosages

Irrational use of injectable antimalarials

Non-adherence to the second line recommendations

Lack of weighing scales

Non-adherence to uncomplicated malaria treatment

Promotion of parasitological malaria diagnosis

Supervision of private facilities.

artemether injections

Supervision of public health facilities.

External quality assessments for malaria diagnosis

Promotion of rational antimalarial use

Dismissal of mixed infection test results

Unknown risks and incorrect dosage schedules of primaquine

Lack of AL follow on treatment and discharge on artesunate injections

Poor injectable artesunate preparation and disposal practices

Lack of weight-based artesunate dosing

Use of prohibited artemether injections

Dismissal of mixed infection test results

Unknown risks and incorrect dosage schedules of primaquine

Non-adherence to *P.vivax* and mixed infection treatments

Lack of AL follow on treatment and discharge on artesunate injections

Non-adherence to severe malaria treatment

Use of prohibited artemether injections

Lack of AL follow on treatment and discharge on artesunate injections

Poor injectable artesunate preparation and disposal practices

Lack of weight-based artesunate dosing
